# Usefulness of a novel drill dilator in removing a migrated biliary plastic internal stent

**DOI:** 10.1055/a-2291-9255

**Published:** 2024-04-09

**Authors:** Takafumi Yanaidani, Tomoaki Matsumori, Masataka Yokode, Yuya Muramoto, Masahiro Shiokawa, Norimitsu Uza, Hiroshi Seno

**Affiliations:** 1 Department of Gastroenterology and Hepatology, Kyoto University Graduate School of Medicine, Kyoto, Japan

A 45-year-old woman referred to hospital for preoperative evaluation of perihilar cholangiocarcinoma ( Fig. 1 ) had multiple biliary plastic internal stents placed previously ( Fig. 2 **a** ). Endoscopic retrograde cholangiography (ERC) was performed to evaluate tumor extent and replace the plastic internal stents. ERC revealed that a nylon-threaded plastic stent (Through & Pass, 7 Fr, 9 cm; Gadelius Medical, Tokyo, Japan) placed in the left hepatic duct (B2) had migrated into the peripheral bile duct ( Fig. 2 **b** ). The nylon threads had entered the common bile duct and the migrated plastic stent could not be removed by pulling them.

**Fig. 1:**
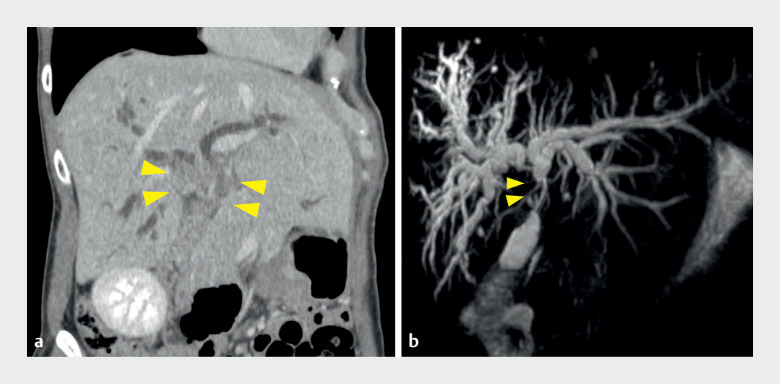
Imaging from the previous hospital. **a** Computed tomography showed cholangiocarcinoma located in the perihilar bile duct (yellow arrows), with dilated intrahepatic bile ducts. **b** Magnetic resonance cholangiopancreatography revealed a hilar biliary stricture (yellow arrows) and dilated intrahepatic ducts.

**Fig. 2:**
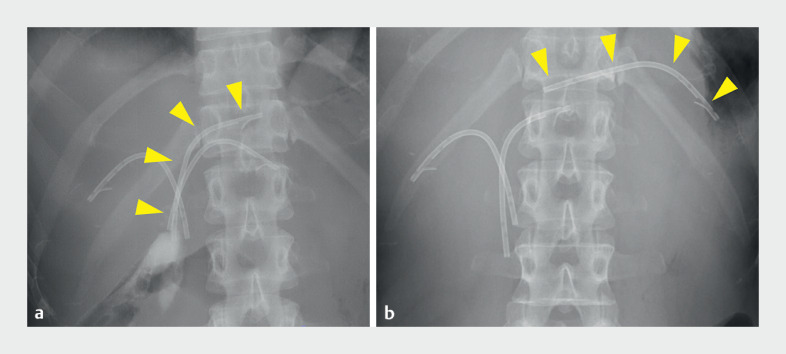
Fluoroscopic images. **a** Multiple biliary plastic stents were placed at another hospital. **b** One plastic stent had migrated into the peripheral bile duct; yellow arrowheads indicate the stent at B2.

Attempts to remove the stent using a stent retriever (Soehendra Stent Retriever; Cook Medical Japan, Tokyo, Japan) and snare catheter (SD-5U-1; Olympus, Tokyo, Japan), failed, breaking and deforming the proximal stent tip in the process ( Fig. 3 ). A guidewire was inserted into the migrated internal stent in an attempt to insert a balloon catheter (REN; Kaneka Co., Inc., Osaka, Japan) into the stent along the guidewire; however, it could not be inserted because of the broken stent tip. Finally, we used a novel 7-Fr drill dilator with a tapered screw-shaped tip (Tornus ES; Olympus) ( Fig. 4 **a** , Video 1 ). Using pushing and clockwise rotation, the dilator was passed through the biliary stricture and easily inserted into the broken stent tip along the guidewire, facilitating successful removal of the stent ( Fig. 4 **b–d** ). New plastic internal stents were placed after tumor evaluation.

**Fig. 3:**
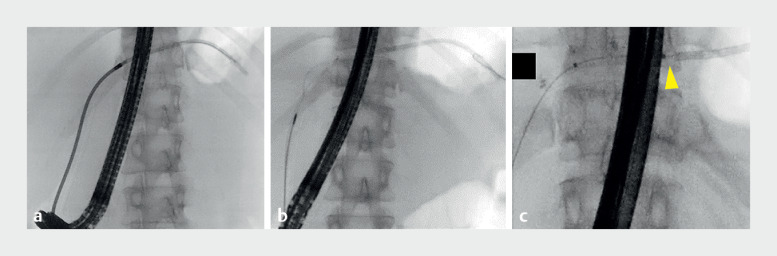
Fluoroscopic images of conventional stent removal methods for a migrated biliary plastic stent. **a** A guidewire was inserted into the stent and a stent retriever was inserted along the guidewire. **b** A snare catheter was used to grasp the migrated stent. **c** Insertion of a balloon catheter (yellow arrowhead: balloon catheter tip) into the stent was challenging due to the broken stent tip.

**Fig. 4:**
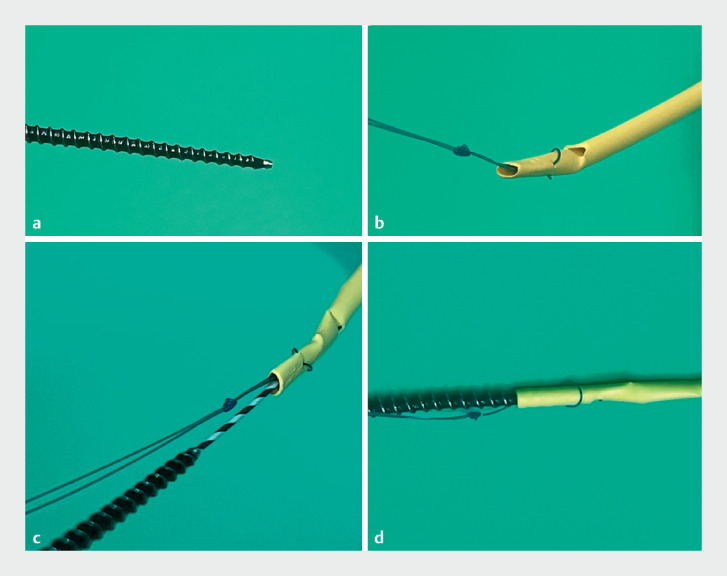
Removal of the biliary plastic stent using a novel drill dilator. **a** Tip shape of the novel drill dilator. **b** Broken and deformed proximal tip of the biliary plastic stent. **c** The guidewire was passed through the stent, and the novel device proceeded along the guidewire. **d** The novel device was inserted into the broken proximal tip of the plastic stent, facilitating successful removal of the stent.

Insertion of the novel drill dilator into the migrated biliary plastic stent and successful stent removal.Video 1

Although there are several methods for removal of biliary stents 1 2 , some cases remain challenging when the stent migrates into the peripheral bile duct. Use of this novel dilator for endoscopic procedures, such as the removal of biliary stents placed across the duodenal papilla, has been reported 3 4 5 . This novel drill dilator has a tapered, sharp, corkscrew tip allowing passage through the biliary stricture and firm grasp of the plastic stent, making it useful for removing migrated biliary internal stents in technically challenging cases.

Endoscopy_UCTN_Code_TTT_1AR_2AZ

## Notes

Endoscopy E-Videos https://eref.thieme.de/e-videos
*E-Videos* is an open access online section of the journal *Endoscopy* , reporting on interesting cases and new techniques in gastroenterological endoscopy. All papers include a high-quality video and are published with a Creative Commons CC-BY license. Endoscopy E-Videos qualify for HINARI discounts and waivers and eligibility is automatically checked during the submission process. We grant 100% waivers to articles whose corresponding authors are based in Group A countries and 50% waivers to those who are based in Group B countries as classified by Research4Life (see: https://www.research4life.org/access/eligibility/ ). This section has its own submission website at https://mc.manuscriptcentral.com/e-videos .
